# Supplementary material for: Rubicon modulates neuroimmune responses following traumatic brain injury
Source: bioRxiv. 2026 Mar 6:2026.03.04.709622. Preprint. [Version 1] doi: 10.64898/2026.03.04.709622 (PMC13001424; doi:10.64898/2026.03.04.709622)
Supplement: 1 [file NIHPP2026.03.04.709622V1-supplement-1.pdf]

bioRxiv preprint doi: https://doi.org/10.64898/2026.03.04.709622; this version posted March 6, 2026. The copyright holder for this preprint (which was not certified by peer review) is the author/funder, who has granted bioRxiv a license to display the preprint in perpetuity. It is made available under aCC-BY 4.0 International license.

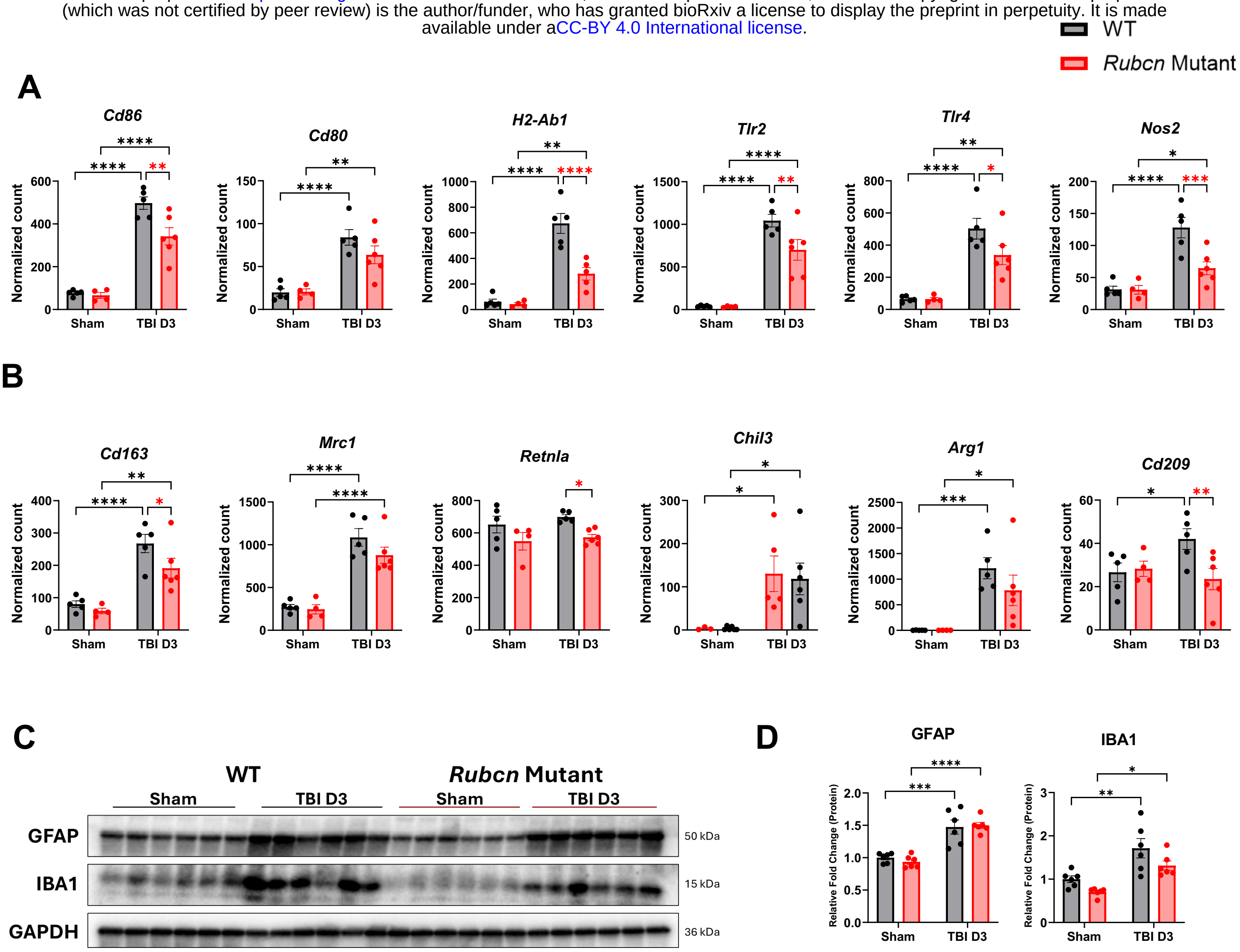

Supplemental Figure 1: Rubicon mutation decreases microglial pro-inflammatory polarization

A Normalized count of pro-inflammatory (M1-like) mRNA in wild-type and *Rubcn*-mutant mice after day 3 (TBI D3) of injury in comparison to sham. **B** Normalized count of anti-inflammatory (M2-like) mRNA in wild-type and *Rubcn*-mutant mice after day 3 (TBI D3) of injury in comparison to sham. **C** Immunoblot comparing ipsilateral cortices from wild-type and *Rubcn*-mutant mice after day 3 of injury to sham. **B** Densitometric quantification of immunoblot in **D**. Bars represent mean±SEM. Sample size (n) for **A-B** were as follows: WT Sham = 5, WT TBI D3=5, *Rubcn* Mut. Sham=4, *Rubcn* Mut. TBI D3=6. Statistical analyses: Two-way ANOVA with post-hoc Uncorrected Fisher's LSD test. Sample size (n) for **C-D** were as follows: WT Sham = 6, WT TBI D3=6, *Rubcn* Mut. Sham=6, *Rubcn* Mut. TBI D3=6. Statistical analyses: Two-way ANOVA with post-hoc Tukey's test. Significance is represented by * p-value<0.05, ** p-value<0.005, *** p-value<0.0005, **** p-value<0.0001.

bioRxiv preprint doi: https://doi.org/10.64898/2026.03.04.709622; this version posted March 6, 2026. The copyright holder for this preprint (which was not certified by peer review) is the author/funder, who has granted bioRxiv a license to display the preprint in perpetuity. It is made available under aCC-BY 4.0 International license.

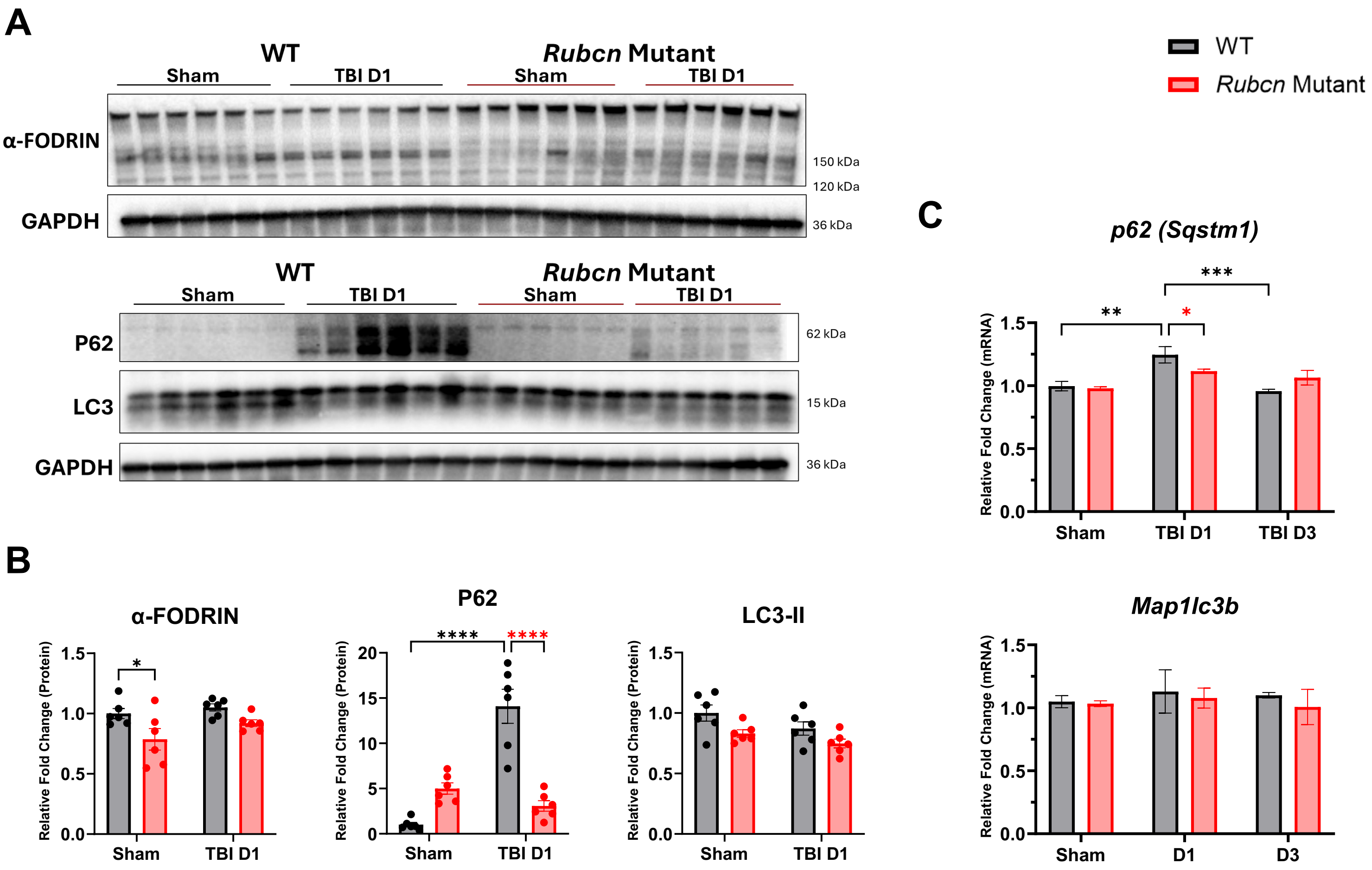

Supplemental Figure 2: Rubicon mutation decreases injury-induced autophagy inhibition

A Immunoblot comparing damage-associated marker (top) and autophagy markers (bottom) in ipsilateral cortices from wild-type and *Rubcn*-mutant mice after 1 day post injury (dpi).**B** Densitometric quantification of immunoblot in **A**. **C** Fold change of mRNA levels of *Sqstm1* and *Map1lc3b* in wild-type and *Rubcn*-mutant mice after 1 and 3 dpi compared to sham. All bars represent mean±SEM. Sample size (n) for **A-B** were as follows: WT Sham = 6, WT TBI D3=6, *Rubcn* Mut. Sham=6, *Rubcn* Mut. D3=6. Sample size (n) for **C** were as follows: WT Sham = 3, WT TBI D3=3, *Rubcn* Mut. Sham=3, *Rubcn* Mut. D3=3. Statistical analyses: Two-way ANOVA with post-hoc Tukey's test and significance represented by * p-value<0.05, ** p-value<0.005, *** p-value<0.0005, **** p-value<0.0001.

bioRxiv preprint doi: https://doi.org/10.64898/2026.03.04.709622; this version posted March 6, 2026. The copyright holder for this preprint (which was not certified by peer review) is the author/funder, who has granted bioRxiv a license to display the preprint in perpetuity. It is made available under aCC-BY 4.0 International license.

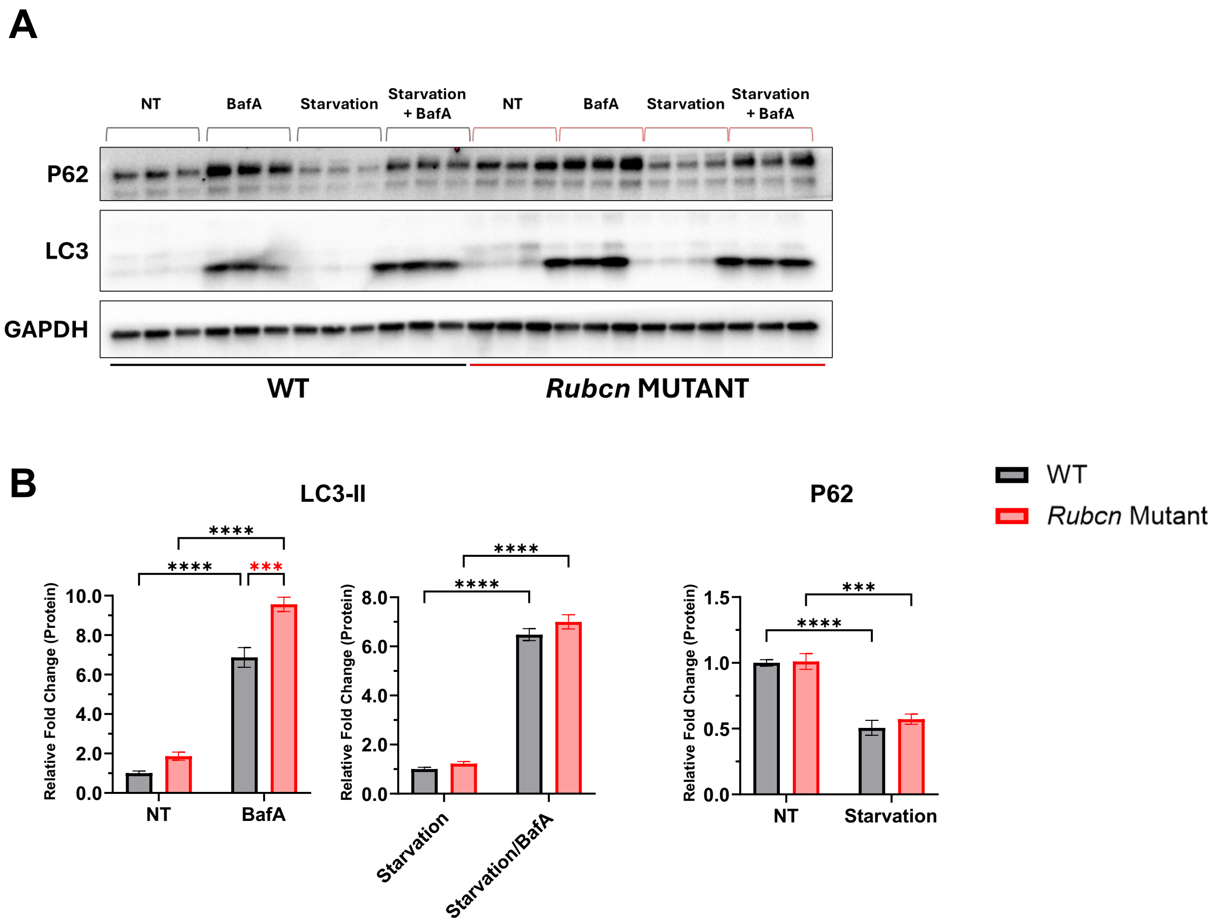

Supplemental Figure 3: Rubicon mutation increases baseline autophagy flux *in vitro*

A Immunoblot of wild-type and *Rubcn*-mutant bone marrow derived macrophages (BMDMs) pre-treated with 50 nM Bafilomycin A (BafA) for an hr. and/or serum-starved (Starvation) for 4 hrs. NT represents no treatment group. **B** Densitometric analysis from **A.** Data represent mean±SEM. Sample size (n) were as follows: n=3 per group. Statistical analyses: Two-way ANOVA with post-hoc Uncorrected Fisher's LSD test and significance represented by * p-value<0.05, ** p-value<0.005, *** p-value<0.0005, **** p-value<0.0001.

bioRxiv preprint doi: https://doi.org/10.64898/2026.03.04.709622; this version posted March 6, 2026. The copyright holder for this preprint (which was not certified by peer review) is the author/funder, who has granted bioRxiv a license to display the preprint in perpetuity. It is made available under aCC-BY 4.0 International license.

A

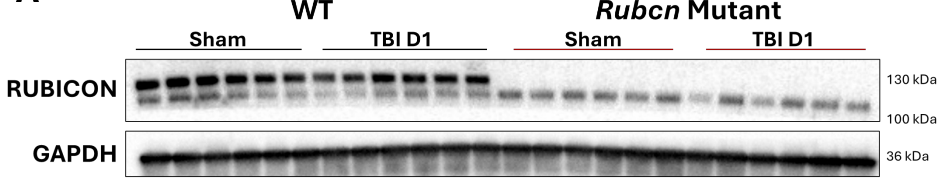

B

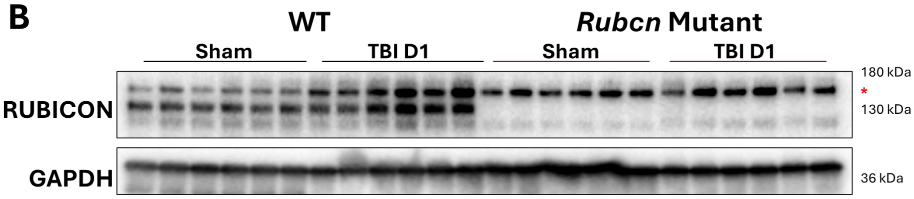

C

Peptide	Positions in Master Proteins	WTRbA	WTRbA	WTRbB	WTRbB	MUTRbA	MUTRbA	MUTRbB	MUTRbB
p1	Q80U62 [35-51]	757897.12							
p2	Q80U62 [79-87]	901011.991	478468.75	297272.9	647483.29				
p3	Q80U62 [103-120]	1533532.72	649298.06	552695.3	503595.54				
p4	Q80U62 [195-204]	589246.797							
p5	Q80U62 [248-264]	515630.44	189056.61		134008.99				
p6	Q80U62 [364-378]	1367169.48	1044234.4	907214	772475.59	350636.6	804352.7	1182071	908300
p7	Q80U62 [389-404]	740187.94		408680.6	429816.09			2138834	548258.7
p8	Q80U62 [550-560]	843720.199	275065.28	353562	131211.96	71575.27		1193284	167100.9
p9	Q80U62 [620-644]		234036.55		183236.68			782318.1	178607.3
p10	Q80U62 [645-666]	2176758	813405.06	1241401	822679.16			1955368	1109239
p11	Q80U62 [768-785]		54991.824	125890.1	62090.446		31876.57	162106.6	70755.47
p12	Q80U62 [872-892]								

D

Accession	Description	Gene Symbol	WT RbA/IgG	MUT RbA/IgG	FC WT/MUT	WT RbB/IgG	MUT RbB/IgG	FC WT/MUT
**Q8K245**	UV radiation resistance-associated protein [OS=Mus musculus]	Uvrag	64.64063487	9.467409597	**6.8277**	51.66830471	79.07346773	**0.653422**
**P51150**	Ras-related protein Rab-7[a] [OS=Mus musculus]	Rab7a	1.110796762	1.481039739	**0.750011**	0.679834887	2.011906463	**0.337906**
**Q6PF93**	Phosphatidylinositol 3-kinase catalytic subunit type 3 [OS=Mus musculus]	Pik3c3	2.173838277	0.726108922	**2.993818**	1.775936206	1.933544679	**0.918487**
**O88597**	Beclin-1 [OS=Mus musculus]	Becn1	5.494405324	1.665575437	**3.298803**	5.340115112	6.68127848	**0.799265**
**Q8BMT4**	Transforming growth factor beta activator LRRC33 [OS=Mus musculus]	Nrros	5.395288987	1.234542762	**4.370273**	3.067699439	0.163886632	**18.71842**

E

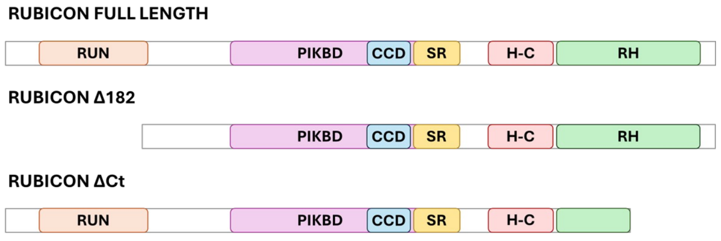

G

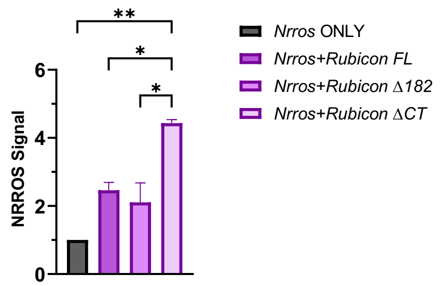

F

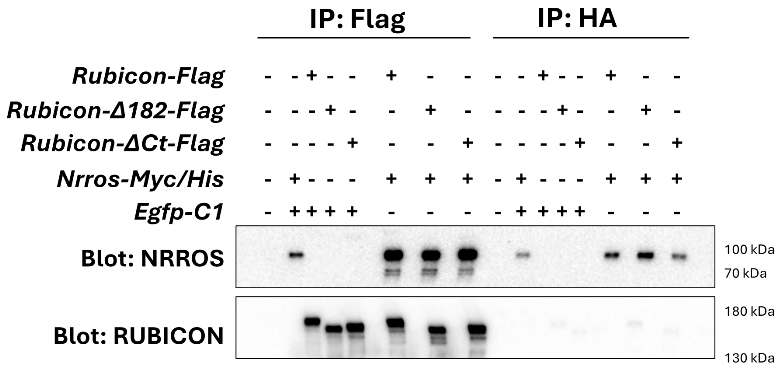

H

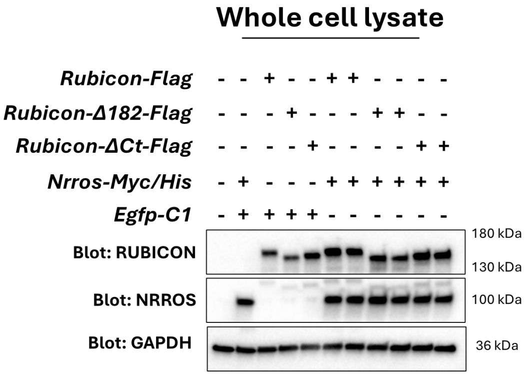

Supplemental Figure 4

bioRxiv preprint doi: https://doi.org/10.64898/2026.03.04.709622; this version posted March 6, 2026. The copyright holder for this preprint (which was not certified by peer review) is the author/funder, who has granted bioRxiv a license to display the preprint in perpetuity. It is made available under aCC-BY 4.0 International license.

Supplemental Figure 4: RUBCN protein interacts with NRROS *in vivo* and *in vitro*. **A** Immunoblot comparing lysates of ipsilateral cortices from wild-type and *Rubcn*-mutant mice probed with Rubicon A (RbA) antibody. **B** Immunoblot comparing lysates of ipsilateral cortices from wild-type and *Rubcn*-mutant mice probed with Rubicon A (RbB) antibody. * represents background signal. **C** Table showing normalized peptide abundance queried for peptides p1 to p12 across the length of RUBCN protein in wild-type and *Rubcn*-mutant immunoprecipitates using RbA and RbB antibodies. n=2 biological replicates for each genotype. **D** Analysis of comparative enrichment of proteins in wild-type over *Rubcn*-mutant immunoprecipitates using RbA and RbB antibodies. **E** Schematic diagram showing full-length and truncated mutants of RUCBN proteins and their protein domains. **F** Immunoprecipitation assay showing lysates of Hek293T cells transfected with indicated plasmids for 24 hrs. and immunoprecipitated with anti-FLAG or anti-HA agarose beads. Immunoprecipitates were subjected to western blot with the indicated antibodies. **G** Fold change of NRROS signal in *Nrros*+*Rubicon(FL, ∆182, or ∆CT*) transfected cells to *Nrros* ONLY transfected cells. Each FLAG IP sample was normalized to corresponding to HA IP sample. **H** Immunoblot of whole cell lysates used in immunoprecipitation assay in **F**. Data represent mean±SEM. Sample size (n) were as follows: n=2 biological replicates. Statistical analyses: One-way ANOVA with post-hoc Tukey's test and significance represented by * p-value<0.05, ** p-value<0.005.
